# DPP4 Gene Polymorphism Associates With Type 2 Diabetes Mellitus in Older Adults

**DOI:** 10.1093/geroni/igaa057.406

**Published:** 2020-12-16

**Authors:** Elisa Alves, Audrey Tonet-Furioso, Izabela D Bastos, Clayton Moraes, Otávio Nóbrega

**Affiliations:** 1 Universidade de Brasília, Brasília, Brazil; 2 Universidade de Brasília, Brasília, Distrito Federal, Brazil

## Abstract

Genetic variations of the dipeptidyl peptidase 4 enzyme (DPP-4) have been associated with glycemic disorders, especially among older adults. The objective of this study was to investigate the association of a single nucleotide polymorphism on the DPP4 gene with the occurrence of type 2 diabetes mellitus in a sample of older community-dwelling patients. Clinical, anthropometric and physiological characteristics as well as biochemical data related to lipidemia, glycemia and hormonal factors and variables related to the lifestyle were analyzed among 338 individuals aged 60 years and older from two general geriatric outpatient clinics in the Federal District, Brazil. Genotypes related to the polymorphism rs3788979 (A/G) of the DPP4 gene of these patients were determined by conventional polymerase chain reaction followed by enzymatic restriction, whereas the serum levels of DPP-4 were assessed by colorimetric immunoassay. Among the results obtained, there was clear variation in the glycemic levels both in terms of blood glucose and glycosylated hemoglobin according to DPP-4 genotypes, with increased levels between homozygotes for the G allele. However, no association was found between genotypes and occurrence of type 2 diabetes mellitus in patients. No other clinical, biochemical or anthropometric variables were influenced by the polymorphism. In our conditions, there was no association between genotypes and DPP-4 levels. Considering that DPP-4 is involved in the metabolism of incretins and directly interferes with glycemia in organisms, our result corroborates that genetic variations of this enzyme can affect glycemic homeostasis, despite not being determinant for the occurrence of type 2 diabetes mellitus.

